# Evaluating the Efficacy of Web-Based Cognitive Behavioral Therapy for the Treatment of Patients With Bipolar II Disorder and Residual Depressive Symptoms: Protocol for a Randomized Controlled Trial

**DOI:** 10.2196/46157

**Published:** 2023-05-19

**Authors:** Gilmar Gutierrez, Callum Stephenson, Jazmin Eadie, Elnaz Moghimi, Mohsen Omrani, Dianne Groll, Claudio N Soares, Roumen Milev, Gustavo Vazquez, Megan Yang, Nazanin Alavi

**Affiliations:** 1 Department of Psychiatry Queen's University Kingston, ON Canada; 2 Department of Psychology Queen's University Kingston, ON Canada; 3 Department of Education Queen's University Kingston, ON Canada; 4 Centre for Neuroscience Studies Queen's University Kingston, ON Canada; 5 OPTT Inc Toronto, ON Canada; 6 Providence Care Center Queen's University Kingston, ON Canada

**Keywords:** bipolar disorder, cognitive behavioral therapy, depression, eHealth, electronic care, internet, mental health, psychotherapy, treatment, web-based therapy

## Abstract

**Background:**

Bipolar disorder (BD) is a highly prevalent psychiatric condition that can significantly impact every aspect of a person’s life if left untreated. A subtype of BD, bipolar disorder type II (BD-II), is characterized by long depressive episodes and residual depression symptoms, with short-lived hypomanic episodes. Medication and psychotherapy, such as cognitive behavioral therapy (CBT), are the main treatment options for BD-II. CBT specific for BD-II involves the recognition of warning signs, potentially triggering stimuli, and the development of coping skills to increase euthymic periods and improve global functioning. However, access to in-person CBT may be limited by several barriers, including low availability, high costs, and geographical limitations. Thus, web-based adaptations of CBT (e-CBT) have become a promising solution to address these treatment barriers. Nevertheless, e-CBT for the treatment of BD-II remains understudied.

**Objective:**

The proposed study aims to establish the first e-CBT program specific for the treatment of BD-II with residual depressive symptoms. The primary objective of this study will be to determine the effect of e-CBT in managing BD symptomatology. The secondary objective will be to assess the effects of this e-CBT program on quality of life and resilience. The tertiary objective will involve gathering user feedback using a posttreatment survey to support the continuous improvement and optimization of the proposed program.

**Methods:**

Adult participants (N=170) with a confirmed diagnosis of BD-II experiencing residual depressive symptoms will be randomly assigned to either the e-CBT and treatment as usual (TAU; n=85) group or the TAU (n=85) control group. Participants in the control group will be able to participate in the web-based program after the first 13 weeks. The e-CBT program will consist of 13 weekly web-based modules designed following a validated CBT framework. Participants will complete module-related homework and receive asynchronous personalized feedback from a therapist. TAU will consist of standard treatment services conducted outside of this research study. Depression and manic symptoms, quality of life, and resiliency will be assessed using clinically validated symptomatology questionnaires at baseline, week 6, and week 13.

**Results:**

The study received ethics approval in March 2020, and participant recruitment is expected to begin in February 2023 through targeted advertisements and physician referrals. Data collection and analysis are expected to conclude by December 2024. Linear and binomial regression (continuous and categorical outcomes, respectively) will be conducted along with qualitative interpretive methods.

**Conclusions:**

The findings will be the first on the effectiveness of delivering e-CBT for patients with BD-II with residual depressive symptoms. This approach can provide an innovative method to address barriers to in-person psychotherapy by increasing accessibility and decreasing costs.

**Trial Registration:**

ClinicalTrials.gov NCT04664257; https://clinicaltrials.gov/ct2/show/NCT04664257

**International Registered Report Identifier (IRRID):**

PRR1-10.2196/46157

## Introduction

Bipolar disorder (BD) is a highly prevalent affective psychiatric condition that affects nearly 2% of the global population [1-5]. It is characterized by chronic mood swings fluctuating from mania or hypomania to depression, which often cause significant impairment both socially and occupationally. The manic or hypomanic component involves 3 days or more of persistently elevated mood, recklessness, grandiosity, decreased need for sleep, reduced ability to concentrate, and irritability [2,4,6-8]. The depressive component involves 2 weeks or more of persistently low mood, anhedonia, poor appetite and sleep, reduced ability to concentrate, agitation or motor slowness, and suicidal ideation [4,8]. There are 4 main types of BD that are differentiated by the type, frequency, and severity of the mood episodes. Bipolar I disorder requires at least one manic episode, whereas bipolar disorder type II (BD-II) is characterized by depressive and hypomanic episodes. Cyclothymic disorder involves the presence of depressive and hypomanic states that do not meet diagnostic criteria and last a minimum of 2 years. Lastly, BD with mixed features is characterized by depressive and elevated moods simultaneously [2,4]. Unlike other types of BD, individuals with BD-II tend to experience longer and more persistent depressive episodes, sometimes with residual depressive symptoms, which are further exacerbated by the negative consequences of their hypomanic episodes [8].

Misdiagnosis and delayed treatment are common for individuals with BD, which can result in serious and sometimes irreversible outcomes. These outcomes can include more frequent and severe mood episodes, intellectual disability, loss of independence, and loss of ability to function [2,4,6,7,9-12]. Although mood-stabilizing medication remains the first line of treatment for BD, the beneficial effects of psychosocial treatments, particularly cognitive behavioral therapy (CBT), are now widely recognized [13]. Furthermore, the use of psychotherapy for the management of BD-II has evolved significantly over the past few decades [8]. CBT targets negative or dysfunctional thought patterns and behaviors through cognitive and behavioral restructuring techniques. These techniques aim to help the individual develop healthier or more balanced thought patterns (cognitive restructuration), which can then be applied in day-to-day life (behavioral restructuration). This can help with reducing psychiatric symptomatology and decreasing depressive episode duration along with increasing euthymic periods and improving global functioning and quality of life [14-20]. Furthermore, CBT specific for BD-II often includes techniques to practice acceptance, recognize warning signs of an upcoming mood episode, recognize potentially triggering stimuli, and develop an action plan with helpful steps and considerations to follow before, during, and after a mood episode [8,14,16,17,19,21,22].

However, several barriers prevent the broader uptake of CBT and other in-person psychotherapy options, including high costs, low availability, long waitlists, stigma against psychiatric interventions, and geographical and physical limitations [11]. Thus, web-based adaptations of traditional in-person CBT (e-CBT) have become promising alternatives due to their lowered cost, time effectiveness, flexibility, privacy, and convenience [14,23-25]. Additionally, e-CBT has been shown to increase help-seeking behaviors among patients and to have similar effectiveness at reducing psychiatric symptomatology as in-person CBT [11,14,26-28]. e-CBT programs often include weekly assignments for the participants and may be either fully self-guided or guided with asynchronous support from an assigned therapist [14]. Despite these benefits over in-person CBT, e-CBT options specific to BD remain significantly understudied and underdeveloped, and very few e-CBT programs have focused on BD-II [15-19,29].

To supplement this gap in the literature and guide clinical practice, this will be an open-label randomized controlled trial studying the effectiveness of a novel e-CBT program specific for BD-II in addition to treatment as usual (TAU; e-CBT + TAU). This program will be developed with the help of individuals with lived experience of BD-II to ensure applicability and accuracy. Thus, the primary objective of this study will be to determine the changes in BD symptomatology (hypomanic and depressive symptoms) of participants receiving e-CBT + TAU compared to participants receiving only TAU. Based on the evidence comparing e-CBT specific for BD to psychoeducation, attention controls, and TAU [16,17,19], this study hypothesizes that adding this e-CBT program to TAU will result in better symptom management than TAU by itself. The secondary objective will be to assess the effects of this e-CBT program on quality of life and resilience. The tertiary objective will involve gathering user feedback using a posttreatment survey to support the continuous improvement and optimization of the proposed program.

## Methods

### Participants

This is the protocol for a randomized controlled trial studying the efficacy of a novel e-CBT program. Participants (N=170) diagnosed with BD-II experiencing residual depressive symptoms will be recruited from the Mood and Anxiety Clinic at Providence Care Hospital, Kingston Health Sciences Center, family physicians, other health care professionals, and self-referrals within Ontario, Canada. Additionally, recruitment efforts will be carried out through flyers in social gathering areas (eg, coffee shops, college campuses), media advertisements, and expert end user referrals. After providing consent to participate in the study, the participants will complete a Mini International Neuropsychiatric Interview 7.0.2 (MINI) with a trained research assistant [30]. The MINI assesses 17 common psychiatric conditions following the diagnostic criteria of the Diagnostic and Statistical Manual of Mental Disorders, 5th edition (DSM-5) [30]. Inclusion criteria include being 18 years or older at the start of the study, a diagnosis of BD-II based on the MINI assessment, a Montgomery-Åsberg Depressive Rating Scale (MADRS) score of mild to moderate depression symptomatology (MADRS score 7-34), competence to consent and participate, ability to speak and read English, and consistent and reliable access to the internet [14,31-33]. Exclusion criteria include acute hypomanic or manic episodes, acute psychosis, severe alcohol or substance use disorder, and active suicidal or homicidal ideation. Additionally, individuals who are currently receiving or have received CBT in the past 6 months will be ineligible to avoid confounding treatment effects.

### Procedures

Eligible participants will be randomly assigned to either a 13-week e-CBT program in addition to TAU (n=85; e-CBT + TAU group) or TAU only (control group; n=85). If interested, participants in the control group will be offered access to the e-CBT program after completing 13 weeks as part of the control group. The e-CBT program will be delivered as an augmentation to TAU through the Online Psychotherapy Tool (OPTT), a secure and interactive platform developed by the principal investigator (PI) [14,34]. TAU will consist of medications, regular physician or clinician visits, referrals, or consultations that are conducted outside of this research study.

### e-CBT Specific for BD-II

Participants in the e-CBT group will receive weekly predesigned web-based modules for 13 weeks. Each module will consist of approximately 30 slides and should take an estimated 45 minutes to complete. Each participant will be assigned a therapist to communicate with them asynchronously through the OPTT platform. In addition, the modules include weekly homework for the participants, which will be submitted directly through OPTT to the assigned therapists. The therapists will then use the OPTT platform to provide personalized feedback using session-specific therapy feedback templates. These feedback templates were developed under the guidance of the PI (NA) to standardize the quality and content of the feedback across different patients and therapists. Furthermore, in previous studies conducted by the PI, these feedback templates have been used to prepare personalized feedback in approximately 15-20 minutes [14,33,35,36].

The proposed e-CBT program will be developed to teach core CBT concepts (ie, the 5-part model, automatic thoughts, the thought record, cognitive and behavioral restructuration) and concepts specific to the management of BD-II [8,14,35,37]. These BD-II–specific concepts include techniques to practice acceptance, recognize warning signs of an upcoming mood episode, recognize potentially triggering stimuli, and develop an action plan with helpful steps and considerations to follow before, during, and after a mood episode [8,14,16,17,19,21]. Additionally, individuals with lived experience of BD-II will be recruited to help in module development and review the modules for applicability and accuracy.

### Training

Research assistants trained in the delivery of CBT and writing feedback will be assigned as therapists for the e-CBT group. The PI, who is an expert in the web-based delivery of psychotherapy and CBT [14,33,35-37], is involved in the training of the therapists to ensure the quality and reliability of the treatment programs. Training will involve weekly workshops and a CBT training video with a quiz at the end. To further ensure that the therapists are well prepared, the co-PI (MY) will closely guide the therapists through their first patient (assigning modules, reviewing homework, writing feedback, and answering questions using the OPTT platform). Furthermore, all therapists will use predesigned session-specific feedback templates to secure the quality of the feedback, which will be reviewed by the co-PI before being sent to the participants. To avoid a conflict of interest, the PI will not be involved in writing or supervising feedback.

### Outcome Evaluation

The primary outcome of this study will be to determine the change in BD symptomatology (hypomanic and depressive symptoms) of participants receiving e-CBT + TAU compared to participants receiving TAU only. This will be measured through clinically validated symptomatology questionnaires, including the MADRS and the Young Mania Rating Scale (YMRS) [31,32]. The secondary outcome will be to assess the effects of this e-CBT program on quality of life and resilience. These metrics will be assessed using the Quality-of-Life Enjoyment and Satisfaction Questionnaire-Short Form (Q-LES-Q-SF) [38] and the 14-item Resilience Scale (RS-14) [39]. All questionnaires will be completed through OPTT at baseline, week 6, and week 13. Subsequent to the treatment period, follow-up assessments will be conducted for all the outcome measures at 3, 6, and 12 months. The tertiary outcome will involve gathering user feedback using a posttreatment survey. This posttreatment survey will be sent to all participants who completed the e-CBT program and will consist of questions related to their experience going through the modules and using the OPTT platform. Also, it will collect any additional feedback and suggestions to support the continuous improvement and optimization of the proposed program.

### Ethics Approval

This study has been reviewed for ethical compliance by the Queen’s University Health Sciences and Affiliated Teaching Hospitals Research Ethics Board (File Number 6028658) in March 2020. Participant data is only accessible to the health care professionals; otherwise, participants will only be identified by their study ID, and all records will be stored on a secure platform. As part of the consent process, participants are informed that they can withdraw from the study at any time and request for their data to be removed from the analysis. The research team will safeguard the data and privacy of the participants to the extent permitted by the applicable laws and duty to report.

OPTT is compliant with the Health Insurance Portability and Accountability Act, Personal Information Protection and Electronic Documents Act, and Service Organization Control-2. Also, to assure provincial and federal privacy and security regulations are met, all servers and databases are hosted in the Amazon Web Service Canada cloud infrastructure. OPTT only collects anonymized metadata used in service quality analysis; no identifiable personal information or IP addresses are collected.

### Data Analysis

Initially, all data will be examined for missing, nonsensical, and outlying variables. Missing data will be treated as missing and not imputed (ie, will be analyzed on a per-protocol basis). However, an intention-to-treat analysis will be conducted to determine the clinical effects of the e-CBT program on participants who dropped out. Based on previous experience with CBT and e-CBT in similar patient populations, the anticipated dropout rate is up to 30% by the end of the treatment or TAU phases [14,33,35,37,40]. Given the likelihood of participant dropout or withdrawal, the study sample has been purposely oversampled to obtain meaningful and statistically significant results at the end of the study. Considering the effect size for e-CBT reported by Todd et al [19] for the reduction of depression symptomatology (effect size=0.44), a significance of P<.05 and a power of 0.80, a sample size of 170 (85 per arm) would be sufficient to detect significant changes from this intervention [14,19,33,35,40,41]. As this is the first study aiming to establish an e-CBT program specific for BD-II, this sample size calculation relies on data from e-CBT specific for BD (all types). It takes into account the established effectiveness of web-based psychotherapy [14].

A 2-tailed significance level of α=.05 will be used for all calculations, except when a Bonferroni correction is needed. Possible differences between program completers and noncompleters will be determined by comparing the respective demographic information using independent sample *t* tests. A 2-by-3 repeated measures ANOVA of the primary (BD symptomatology: MADRS and YMRS) and secondary (Q-LES-Q-SF and RS-14) outcomes will be conducted to compare the effects of e-CBT + TAU to TAU only throughout the 13-week treatment course (0-, 6-, and 13-week assessment points). Variables associated with outcome measures will be determined using linear regression (continuous outcomes) and binomial regression analysis (categorical outcomes). The data collected from the posttreatment survey will be analyzed qualitatively with 2 reviewers to identify common themes and specific areas for improvement. All statistical analyses will be conducted using SPSS Statistics for Mac, version 28 (IBM Corp).

## Results

The literature comparing the efficacy of e-CBT specific for BD to psychoeducation, attention controls, and TAU has shown that e-CBT specific for BD is effective at reducing BD symptomatology (depression and mania or hypomania symptoms) and improving quality of life [16,17,19]. Thus, this study hypothesizes that adding this e-CBT program to TAU will result in better symptom management (reduction of BD symptomatology and improvement of quality of life) than TAU by itself. The study received notice of funding acceptance through the Queen’s University Department of Psychiatry Internal grant in December 2019. Participant recruitment will begin in February 2023 using targeted advertisements and physician referrals (see Methods). Complete data collection and analysis from all phases are expected to conclude by December 2024. All procedures and outcomes have been and will be reported using the CONSORT (Consolidated Standards of Reporting Trials) and TIDieR (Template for Intervention Description and Replication) Report Guidelines [42]. This study has been registered through the ClinicalTrials.gov system (NCT04664257).

## Discussion

This is a protocol for an RCT that aims to establish the first e-CBT program for the management of BD-II with residual depressive symptoms. This program will cover helpful coping techniques to increase euthymic periods and improve global functioning by applying the widely validated principles of CBT [14-20]. Based on literature evidence, previous clinical trials conducted by NA, and the use of a validated therapy approach, this study hypothesizes that this novel e-CBT program will have similar effectiveness as face-to-face CBT for the management of BD-II with residual depressive symptoms [14,33,35-37].

The management of BD-II involves the use of mood-stabilizing medications and psychosocial interventions [13]. Thus, given the proven efficacy of CBT for a variety of psychiatric conditions, the development of CBT for the management of BD-II has become a growing area of research [8]. CBT for BD-II usually involves face-to-face sessions with a therapist and covers techniques to practice acceptance, recognize warning signs of an upcoming mood episode, recognize potentially triggering stimuli, and develop an action plan [14-20]. However, access to in-person CBT faces significant barriers, including high costs, low availability, long waitlists, stigma against psychiatric interventions, and geographical and physical limitations [11]. Also, despite advances in the development and validation of web-based adaptations of CBT for other psychiatric conditions [14,15,20], e-CBT options specific for the treatment of BD-II remain understudied [15-19]. This presents an important gap in the literature with potentially detrimental consequences for individuals with this condition.

The strengths of this novel e-CBT program and of this study include the use of a validated approach for the delivery of interactive, predesigned web-based modules with the asynchronous guidance of a therapist through the OPTT platform [14,33,35-37]. Also, by taking advantage of the web-based medium, this program has the potential to improve accessibility, cost, time effectiveness, flexibility, privacy, and convenience. For instance, previous studies have shown that asynchronous psychotherapy, through the use of predesigned web-based modules, allows therapists to deliver care to 3-4 patients in the same time as a traditional hour-long in-person session [14,33,35-37]. Furthermore, using the OPTT platform, this study will be able to reliably collect user analytics, which allows for the improvement and continuous optimization of e-CBT program delivery. Additionally, by comparing this proposed program to a validated therapeutic approach (face-to-face CBT for the management of BD-II) [14-20], the design of this study should provide helpful evidence for the validation of e-CBT for the management of BD-II with residual depressive symptoms.

Moreover, the knowledge dissemination plan for this study includes the publication of the findings in a peer-reviewed academic journal. The findings will also be presented broadly through academic meetings and conferences. Thus, this plan ensures the commitment to continuing education in the relevant research areas. Also, collaboration with the Canadian enterprise OPTT Inc. will enhance the dissemination and application of knowledge. Additionally, by providing this service to clinicians, the care capacity for accessible, affordable, and high-quality mental health care could be more rapidly scaled across Canada.

Though this study has some potential limitations that mainly stem from the web-based mode of delivery of this program, the main limiting factor is that participants would need a reliable internet connection and sufficient technology literacy to participate. In turn, this could introduce biases in participant demographic characteristics. To account for this, this study will analyze demographic factors through multiple sample *t* tests to identify the impact of these factors on treatment outcomes. Additionally, the design of this study is limited by the lack of therapist and client blinding. However, due to the delivery mode and design of the program, it is not possible to conceal client allocation. Furthermore, web-based psychotherapy is often characterized by significant dropout rates, with some studies reporting a dropout rate of up to 30% by the end of the treatment or TAU phases [14,33,35,37,40]. Hence, this study is intentionally oversampling to account for this potential high dropout rate without compromising the statistical power of this study.

In conclusion, this study recognizes the significant potential of web-based psychotherapy as a tool that can lower costs and increase capacity and accessibility in mental health care, as well as the lack of available web-based psychotherapy options for individuals with BD-II. Therefore, the results of this study aim to fill an important gap in the literature through the development, implementation, and validation of a novel e-CBT program for the management of BD-II with residual depressive symptoms. Additionally, these results aim to inform clinical practice and health care policy to support the widespread implementation of web-based psychotherapy, with potential benefits to the care capacity and performance of the health care system.

## Appendix

Multimedia Appendix 1 Peer review report by Providence Care Centre Research Innovation Grant (Kingston, Canada).

## Abbreviations

BD: bipolar disorder
BD-II: bipolar disorder type II
CBT: cognitive behavioral therapy
CONSORT: Consolidated Standards of Reporting Trials
e-CBT: web-based adaptations of cognitive behavioral therapy
MADRS: Montgomery-Asberg Depressive Rating Scale
MINI: Mini International Neuropsychiatric Interview 7.0.2
OPTT: Online Psychotherapy Tool
PI: principal investigator
Q-LES-Q-SF: Quality-of-Life Enjoyment and Satisfaction Questionnaire-Short Form
RS-14: 14-item Resilience Scale
TAU: treatment as usual
TIDieR: Template for Intervention Description and Replication
YMRS: Young Mania Rating Scale

## Data Availability

Data sharing is not applicable to this paper as no data sets were generated or analyzed yet for the preparation of this protocol manuscript. Though, the data sets generated and analyzed in the randomized controlled trial will be available from the corresponding author on reasonable request.
