# Supplementary material for: Evaluating the Efficacy of Web-Based Cognitive Behavioral Therapy for the Treatment of Patients With Bipolar II Disorder and Residual Depressive Symptoms: Protocol for a Randomized Controlled Trial
Source: JMIR Res Protoc. 2023 May 19;12:e46157. doi: 10.2196/46157 (PMC10238953; doi:10.2196/46157)
Supplement: Multimedia Appendix 1 [file resprot_v12i1e46157_app1.pdf]

Providence Care

ALAVI N/VAZQUEZ G et al. **Score: 4.0 (Averaged score, 2 reviewers)**
Funding requested: $60,000 (24 months).
DECISION: Likely to be funded after addressing comments below.

Title: Delivering Electronic Cognitive Behavioural Therapy to Patients with Bipolar Disorder and Residual Depressive Symptoms

Title: Delivering Electronic Cognitive Behavioural Therapy to Patients with Bipolar Disorder and Residual Depressive Symptoms **Brief Summary:** The proposal aims to evaluate feasibility and effectiveness of e-CBT delivered via Online Psychotherapy Tool (OPTT) for treatment of BAD-II with residual depressive symptoms, and also analyze social, cultural, and personal factors affecting patients' experience. The proposal aims for 80 patients with of BAD-II with residual depressive symptoms randomized to e-CBT or in-person group therapy for 12 weeks, MADRS/YMRS/CGI-BP-M questionnaires at baseline, 6, 12 weeks will be sued for outcome measurement. Focus groups will be used for examining personal/social/cultural factors.

Strengths:

1.The proposal has brought together an excellent team of investigators with the necessary expertise in mood disorders, online CBT, and clinical trials to successfully carry out the project.

2.The topic is an understudied but important area for subjects with BAD, a substantial proportion of whom suffer from depressive/residual depressive symptoms during any given year.

3.The proposal has a quantitative and qualitative aspect to examine various pertinent factors related to online CBT for BAD-II with residual depressive symptoms.
4.The project is a collaboration with in-kind support from OPTT.

Weaknesses:

1.It is not clear whether BAD-II, current episode depressed vs remitted depression with residual symptoms is the inclusion criteria. The severity cutoff on MADRS at baseline and the residual symptoms(e.g. cognition, sleep) are not described. It is not clear if subjects who have previously had psychotherapy (CBT) would be allowed to participate. For the control arm, the nature of group therapy(e.g. ? CBT) is not described.

2.The power calculation is based on an effect size of 0.9, correlations of r=0.09 and p=0.001. The effect size estimates are on the higher side as the efficacy would be in the moderate effect size range rather than large (0.9). There is no mention of expected drop-out rate/sample size adjustments/ analysis strategy for dropouts (e.g. last subject carried forward/MCMC methods, etc.) which will be an important issue.

Providence Care

3. The challenges with delivering e-CBT in other contexts (e.g. maintaining motivation for completion in a virtual environment, etc.) could be described with approaches in this proposal to deal with these challenges effectively.

4. The proposal has outstanding investigators with expertise on the quantitative side and might benefit from including someone with specific expertise on the qualitative side.

5. BUDGET: Overall, seems reasonable. Adjust Focus group down to $1,500.
